# Biosafety and efficacy evaluation of a biodegradable magnesium-based drug-eluting stent in porcine coronary artery

**DOI:** 10.1038/s41598-021-86803-0

**Published:** 2021-04-01

**Authors:** Jinzhou Zhu, Xiyuan Zhang, Jialin Niu, Yongjuan Shi, Zhengbin Zhu, Daopeng Dai, Chenxin Chen, Jia Pei, Guangyin Yuan, Ruiyan Zhang

**Affiliations:** 1 Department of Cardiology, RuiJin Hospital, School of Medicine, Shanghai Jiao Tong University, Shanghai, 20025 People’s Republic of China; 2 National Engineering Research Center of Light Alloy Net Forming and Key State Laboratory of Metal Matrix Composites, Shanghai Jiao Tong University, Shanghai, 200240 People’s Republic of China

**Keywords:** Interventional cardiology, Biomedical materials

## Abstract

Although the drug-eluting stent (DES) has become the standard for percutaneous coronary intervention (PCI)-based revascularization, concerns remain regarding the use of DES, mainly due to its permanent rigid constraint to vessels. A drug-eluting bioresorbable stent (BRS) was thus developed as an alternative to DES, which can be absorbed entirely after its therapeutic period. Magnesium (Mg)-based BRSs have attracted a great deal of attention due to their suitable mechanical properties, innovative chemical features, and well-proven biocompatibility. However, the primary disadvantage of Mg-based BRSs is the rapid degradation rate, resulting in the early loss of structural support long before the recovery of vascular function. Recently, a new type of patented Mg–Nd–Zn-Zr alloy (JDBM) was developed at Shanghai Jiao Tong University to reduce the degradation rate compared to commercial Mg alloys. In the present investigation, a poly(d,l-lactic acid)-coated and rapamycin eluting (PDLLA/RAPA) JDBM BRS was prepared, and its biosafety and efficacy for coronary artery stenosis were evaluated via in vitro and in vivo experiments. The degree of smooth muscle cell adhesion to the PDLLA/RAPA coated alloy and the rapamycin pharmacokinetics of JDBM BRS were first assessed in vitro. JDBM BRS and commercial DES FIREHAWK were then implanted in the coronary arteries of a porcine model. Neointimal hyperplasia was evaluated at 30, 90, and 180 days, and re-endothelialization was evaluated at 30 days. Furthermore, Micro-CT and optical coherence tomography (OCT) analyses were performed 180 days after stent implantation to evaluate the technical feasibility, biocompatibility, and degradation characteristics of JDBM BRS in vivo. The results show the ability of a PDLLA/RAPA coated JDBM to inhibit smooth muscle cell adhesion and moderate the drug release rate of JDBM BRS in vitro. In vivo, low local and systemic risks of JDBM BRS were demonstrated in the porcine model, with preserved mechanical integrity after 6 months of implantation. We also showed that this novel BRS was associated with a similar efficacy profile compared with standard DES and high anti-restenosis performance. These findings may confer long term advantages for using this BRS over a traditional DES.

## Introduction

Strategies for the treatment of coronary artery disease (CAD) have made significant progress in the past decades. The first performed balloon angioplasty in 1977 changed the landscape of CAD treatment. Bare metal stent (BMS) heralded the second revolution as a means to conquer the drawbacks of balloon angioplasty, including acute vessel recoil, late constrictive remodeling, and diffuse restenosis. Drug-eluting stents (DES), the third revolution in interventional cardiology, have now become the gold standard in percutaneous myocardial revascularization, as DES have been demonstrated to reduce the in-stent restenosis rate and incidence of major cardiac adverse events (MACE) by inhibiting the proliferation of smooth muscle cells compared with BMS^1,2^. However, concerns remain regarding the use of DES, mainly due to its permanent rigid constraint to vessels and consequences like the development of late stent thrombosis (ST)^3–5^. In addition, other issues have been identified, including the reduction of side-branch flow, abnormal vasomotion because of the permanent metallic caging, and interference with future surgical revascularization^6–8^.

A drug-eluting bioresorbable stent (BRS) was thus developed as an alternative to DES. These stents provide temporary support to the vessel wall and can be fully resorbed after the vessel has regain edits physiological function^9–13^. The first generation of poly-l-lactic acid (PLLA) based stent (ABSORB, Abbott Vascular) showed promising results at 1-year post-operation. However, it has had several limitations, including increased strut thickness and crossing profile and relatively low resistance to overexpansion, which originates from its poor mechanical properties. According to long-term reports, these weaknesses caused an increased risk of ST and target vessel myocardial infarction, and consequently, Abbott Vascular opted to halt production^14^. Moreover, PLLA-based BRS lacks radiopacity, resulting in a more frequent need for intravascular imaging modalities to achieve appropriate device deployment.

The new generation of magnesium (Mg)-based BRS has attracted significant attention recently. These stents have suitable mechanical properties and radiopacity, allowing them to circumnavigate the drawbacks of PLLA polymer stents. In contrast to PLLA BRS, Mg shows better radial force, pushability, and trackability. At the same time, the degradation products, being mainly Mg ions, have demonstrated good biocompatibility^15^. Historically biodegradable Mg-based materials have been used in therapeutic medicine in different areas, including wound closure, dental procedures, and cardiovascular surgery. The biocompatibility of Mg-based BRS devices in vascular tissue has been demonstrated in porcine coronary arteries with rapid endothelialization and low inflammatory response^16^. However, the primary disadvantage of Mg-based BRS is its high corrosion rate with complete biodegradation within 3 months; ideal BRS should have adequate radial support for a period of about 6 months to prevent recoil and constrictive remodeling.

Fortunately, the in vivo degradation process of Mg-based BRS could be regulated by alloy design and the adoption of a bioactive coating. Recently, a new type of patented Mg–Nd–Zn-Zr alloy (Jiao Da BioMg, denoted as JDBM) was developed at Shanghai Jiao Tong University^17,18^. In this Bio-Mg alloy series, Nd was selected as the main alloying element, accompanied by the micro-alloyed Zn and Zr. Nd is a light and rare earth element showing little cytotoxicity. Adding Nd to Mg has already demonstrated a significant strengthening of Mg-Nd binary alloys^19^ and may considerably reduce galvanic corrosion between the Mg matrix and second phases^17,20^. As a result, the novel JDBM material possesses excellent mechanical properties, good biocompatibility, a slower corrosion rate, and a more uniform degradation behavior over many commercially available Mg alloys, such as AZ31 and WE43^18^.

Aiming to further reduce the degradation rate of JDBM alloy and restore the physiological function of vessels, in the present study, we designed a type of rapamycin coating-poly(d,l-lactic acid), denoted as PDLLA/RAPA. We prepared it successfully on JDBM alloy disk samples as well as stents. The inhibitory effects on smooth muscle cells and pharmacokinetics were studied in vitro, and the in vivo biosafety and efficacy were evaluated using a porcine coronary arteries model.

## Methods

### Materials and samples preparation

The composition and preparation process of JDBM alloy can be found in Ref.^18^. Disk samples of Ø15 × 3 mm were cut from JDBM extruded bar for the in vitro cell adhesion experiment. After being ground to 3000 grit with SiC paper, the samples were immersed in Hydrogen Fluoride (HF) to form MgF_2_ film for lower degradation of JDBM, and then the PDLLA/RAPA coating was prepared on the disk samples using the same method in Ref.^21^. For stent preparation, a JDBM mini-tube with an outer diameter of 3 mm was prepared, and the stents were laser-carved from the mini-tube in MicroPort Medical Co., Ltd (Shanghai, China). The detailed process can be found in Ref.^22^. After electrochemical polishing, the stents were immersed in HF as mentioned above, and then the PDLLA/RAPA coating was prepared on these stents using the same method in Ref.^23^. The stents finishing preparation were denoted as JDBM BRS. As a control, the same structured 316 L stents (SS) loaded with PDLLA/RAPA coating were also prepared using the same method (SS BRS).

### In vitro cell adhesion

Rat thoracic aorta smooth muscle cell lines (A7r5) purchased from Cell Bank (Chinese Academy of Sciences, China) were selected to evaluate the cell adhesion status on PDLLA/RAPA coated JDBM. The cells were cultured in Dulbecco's Modified Eagle Medium (DMEM, Gibco, CA, USA) and supplemented with 1% streptomycin/penicillin and 10% fetal bovine serum (FBS, Gibco, CA, USA) in a cell incubator with 37 °C humidified atmosphere containing 5% CO_2_.

To study the effects of the coating on A7r5 cells, we prepared three types of disk samples, including PDLLA/RAPA coated JDBM (PDLLA/RAPA), PDLLA coated JDBM (PDLLA), and JDBM only immersed in HF without any polymer coating (HF-JDBM) disk samples. These samples were put in 24-well plates, and every well was supplemented with 1 mL 10,000/mL cell suspension. The samples and cell suspension were co-incubated for 1 to 3 days, then the samples were cleaned twice using PBS, and every well was stained with 200 μL calcein acetoxymethyl ester (Calcein-AM, Sigma, MO, USA). Fifteen minutes later, the samples were cleaned with PBS again and then transferred onto slides. The morphology and quantity of cells were observed by inverted fluorescence microscopy (IX 71, Olympus, Japan) and analyzed using Image J software (NIH, MD, USA). A negative control group (NC) where cells were incubated in blank wells was also set.

### In vitro pharmacokinetics

#### Standard curve of rapamycin

A standard curve of rapamycin should be measured to calibrate the drug release. A 100 μg/mL solution was prepared by dissolving 10 mg rapamycin with acetonitrile. The solution was then diluted to a rapamycin standard solution with concentrations of 50 μg/mL, 25 μg/mL, 12.5 μg/mL, 5 μg/mL, 2.5 μg/mL, 1 μg/mL, 0.5 μg/mL, and 0.1 μg/mL. The absorbance of rapamycin in gradient standard solution was measured by a UV/Vis spectrophotometer (Thermo Spectronic Genesys 10, MA, USA). Finally, the standard curve of rapamycin was obtained by fitting the correlation between absorbance and concentration.

#### Rapamycin pharmacokinetics

To understand the pharmacokinetics of the real stents made by PDLLA/RAPA coated JDBM, the JDBM BRS was immersed in phosphate-buffered saline containing 0.5 v/v% Tween 20 (PBST) and then placed in a shaker with a speed of 80 r/min at 37 ℃. At regular intervals, 2–5 mL PBST was collected and replaced with the same volume of fresh PBST. After centrifuging the collected PBST at a speed of 13,000 r/min, the supernatant was collected to measure the absorbance by UV/Vis spectrophotometry (Thermo Spectronic Genesys 10, MA, USA). Drug concentration and accumulative release were calculated according to the standard curve of rapamycin. The rapamycin pharmacokinetics curves were drawn and analyzed. The pharmacokinetics curve of SS BRS was tested as a control to reveal the impact of JDBM degradation on drug release.

### Porcine study

Animal experiments were conducted according to the standard protocols, animal welfare regulations, and the institutional guidelines of Shanghai Jiao Tong University School of Medicine and the Regulations for Practice of Experimental Animals (issued by Scientific and Technical Committee, PR China, 1988). We confirm that the study was carried out in compliance with the ARRIVE guidelines. All the procedures described were performed with the authorization of the Animal Use and Care Committee of Shanghai Jiao Tong University School of Medicine (approval number: SYKX-2008-0050). The protocol was approved by the institutional ethics committee of Ruijin Hospital (ID: 2020-152), Shanghai, China.

Chinese domestic porcine obtained from Shanghai Agricultural College (Shanghai, China) with a bodyweight between 55 and 90 kg were fed with a standard laboratory chow diet for 7 days. The JDBM BRS and the control stent (FIREHAWK, MicroPort Medical, Shanghai, China) were then implanted into coronary arteries under digital subtraction angiography (INNOVA 2100, GE, USA) with the method as reported previously^24^ and followed for 30, 90, and 180 days. Briefly, arterial access was achieved by surgical exposure of the right common iliac artery with a 20 G puncture needle (Terumo, Tokyo, Japan). Coronary arterial angiography was then performed after intra-arterial administration of heparin (100 IU/kg), and the diameter of the coronary artery was measured with quantitative coronary angiographic analysis (QCA). Stents were implanted mainly in two major branches of the coronary artery: the left anterior descending and the right coronary artery. The balloon was inflated to 12 atm with a stent vessel ratio of 1.1:1. The arteriotomy and dermal layers were sutured after the catheter, wire, and sheath were removed. The stenting procedure was always performed by the same investigators. Three days before the procedure and throughout the following period, all animals received 100 mg Aspirin and 75 mg Clopidogrel via daily administration.

### Quantitative coronary angiography

Angiography was performed before and after interventions and at 30, 90, and 180 days angiographic follow-up using identical projections and analyses. Offline quantitative measurements of the operation were performed with a computerized edge-detection quantitative coronary angiographic analysis software QAngio XA 7.2 (Medis Medical Imaging System BV, Leiden, the Netherlands) by an observer blinded to the study with the method as reported previously^25^. After calibration with the outer diameter of the contrast-filled catheter, the minimum lumen diameter in different groups was determined in the appropriate frame.

### Optical coherence tomography (OCT) analysis

Optical coherence tomography (OCT) was performed at 180 days follow-up using the OCT imaging system C7-XR with a catheter diameter of 2.7F (Light Lab Imaging, St. Paul, MN, USA) in experimental animals with the method as reported previously^26^. The entire length of the stent was imaged with an automatic pullback device acquired at 20 mm/s. To remove blood from the imaging site, a contrast solution was infused into the coronary artery during the retracement. All OCT frames were digitally stored. Cross-sectional OCT images were analyzed at 0.2 mm intervals (every frame) using validated software (Light Lab Imaging, St. Paul, MN, USA) based on expert consensus. The lumen and stent contours of each cross-section were delineated semiautomatically to measure the area of the lumen and stent. The absence of definite neointima over the stent strut was defined as an uncovered stent strut identified by experienced observers. Distances between the lumen and struts were calculated automatically to determine malposition. Strut malposition was determined when the distance was larger than the sum of strut thickness plus polymer thickness (86 μm)^27^. Non-apposed stent struts were included in the analysis for strut coverage but excluded in the analysis for malposition in accordance with previous research^28^. Neovascularization was defined as a signal-poor hole or tubular structure with a diameter of 50–300 µm present on at least three consecutive frames^29^. Thrombus was defined as an irregular mass protruding into the lumen or intraluminal mass with signal-free shadowing unconnected to the luminal surface^30^.

### Micro-CT analysis

Micro-CT scanning was performed to acquire a whole set of raw data along the entire length of the stent sample, and to display the in vivo degradation characteristics of the stent in the porcine coronary arteries after 30, 90, and 180 days of stent implantation (SkyScan 1176, Kontich, Belgium). The long axis was aligned perpendicular to the axis of the X-ray beam. Constant settings for X-ray energy and image capture were maintained. The obtained projection images were processed with the NRecon image reconstruction software V1.4.4 (Kontich, Belgium) using a convolution and back-projection algorithm to produce a stack of 8-bit BMP images, each one of them representing a slice of the sample. Finally, a full three-dimensional (3D) model and animation were generated for visualization of the scaffold.

### Scanning electronic microscopy (SEM) observation

Animals were sacrificed at 30 days post-implantation, and the stented arterial segments were examined *en face* by SEM for analysis of potential stent thrombosis incidence with the method as reported previously^24^. The stented arterial was first flushed with PBS for 1 min, followed by a gentle flush with 10% buffered formalin for 30 s. Samples were further fixed with 2.5% glutaraldehyde in 0.1 M sodium cacodylate buffer overnight and washed thrice with cacodylate buffer. Post-fixation was completed with 1% osmium tetroxide in 0.1 M cacodylate buffer, then serially dehydrated with ethanol (30, 50, 70, 90, 95, and 100%), and critical point drying with CO_2_. After drying, samples were gold sputtered and visualized under SEM (Quanta 250, FEI, OR, USA). The images of the stented artery cross-sections were analyzed, and regions of interest were imaged at incremental magnifications.

### Histological analysis

All animals were sacrificed by potassium chloride injection, and the stented arterial segments, obtained at 30, 90, and 180 days, were processed for histological examination. Samples were dehydrated in a graded series of ethanol and embedded in methylmethacrylate plastic. After polymerization, the center section of each stent was cut on a rotary microtome (EXAKT, Norderstedt, Germany) into 10 μm thick sections and stained with Masson. An experienced pathologist, who was blinded to the groups, performed all histological analysis with the method reported previously^25^.

### Statistics

Statistical analyses were performed with the method as reported previously^31^. Continuous variables are presented as mean ± standard deviation (SD), and categorical variables are presented as counts and percentages. Statistically significant differences over time in the same treatment group or among different treatment groups at a single time point were determined by one-way analysis of variance (ANOVA), followed by two-tailed Student's *t*-tests. Statistical analyses were performed with SPSS 17.0 (IBM, Armonk, NY, USA). Statistical significance was assumed for *P* values < 0.05.

## Results

### In vitro cell adhesion

**Figure 1:**
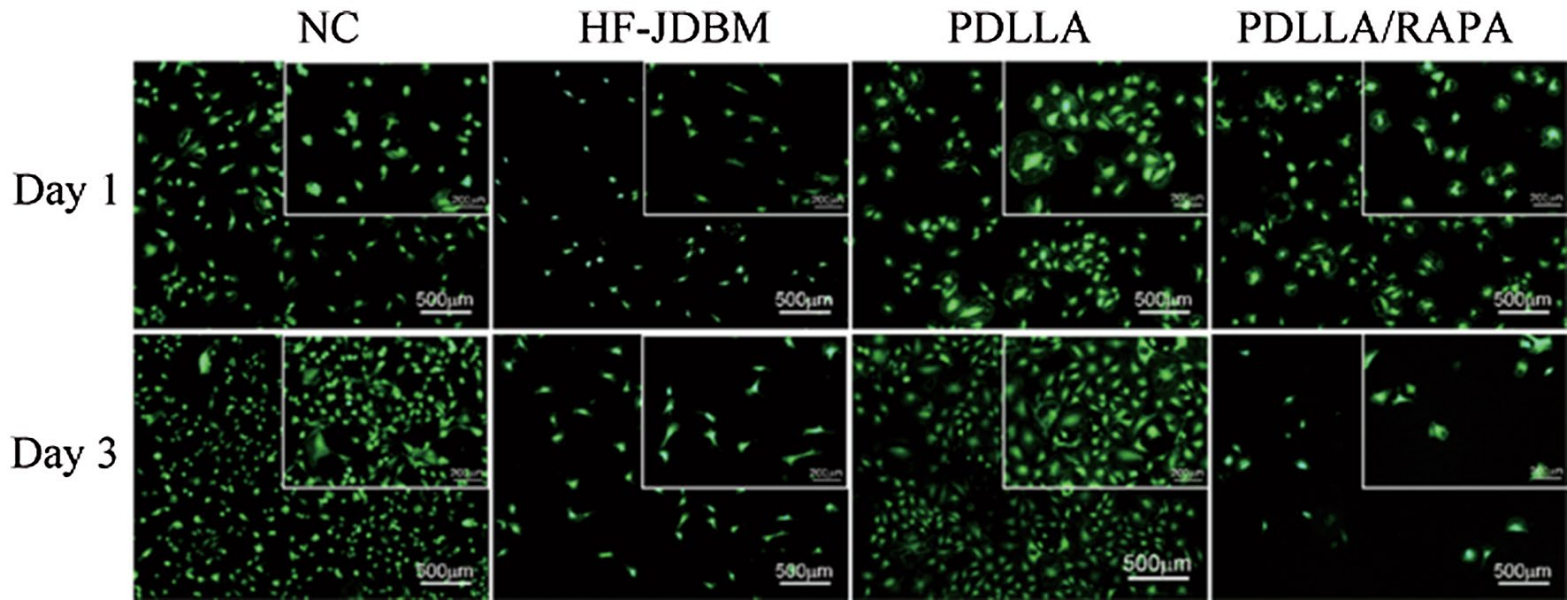
The adhesion morphologies of A7r5 cells on HF-JDBM, PDLLA coated JDBM, PDLLA/RAPA coated JDBM, and negative control (NC) for 1 and 3 days (Stained with Calcein-AM, and green signals represent live cells).

**Figure 2:**
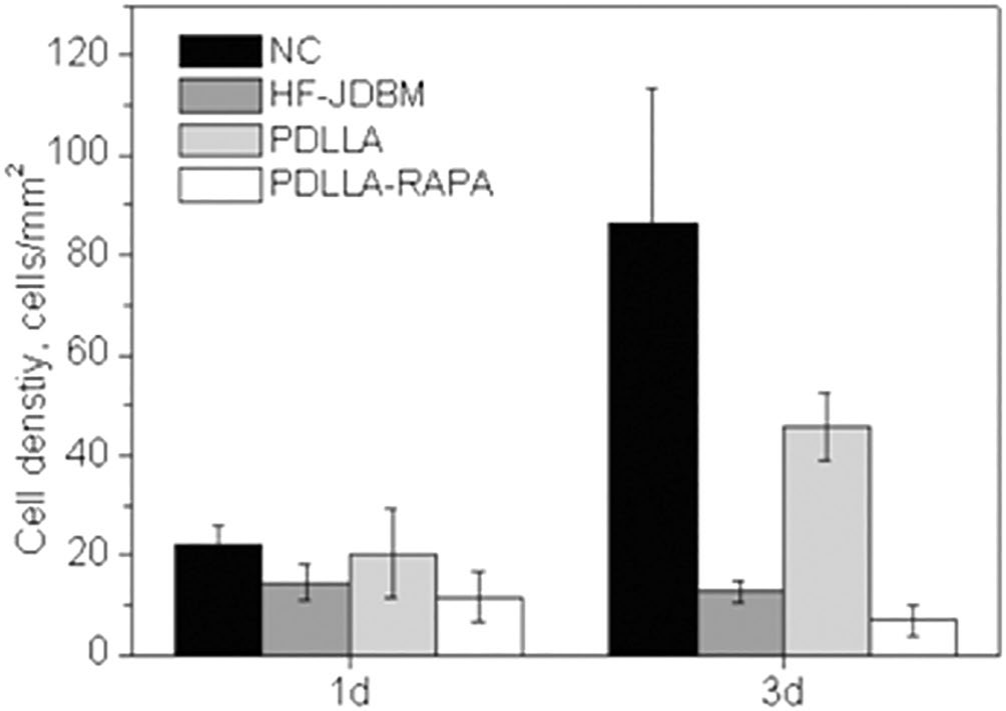
Statistics of A7r5 cell densities on various samples (Five fields are counted for each group and the results are presented as mean ± SD).

The adhesion states of smooth muscle cells on PDLLA/RAPA-coated JDBM, PDLLA coated JDBM, HF-JDBM samples, and Negative control (NC) are shown in Fig. 1, and the cell densities were counted as shown in Fig. 2. After incubated for 1 day, there was no apparent difference in cell density among the four groups, and only the PDLLA/RAPA group showed a slightly lower cell density. However, after incubation for 3 days, four groups displayed diversity. The smooth muscle cell density increased 4 and 2.5 times in the NC and PDLLA groups, respectively, while there was no evident change in the HF-JDBM group. Conversely, the cell density decreased in the PDLLA/RAPA group. The results of cell adhesion indicated that by loading RAPA, PDLLA/RAPA coating on JDBM could inhibit the proliferation of smooth muscle cells effectively. This was realized by controlling the rapamycin release.

### In vitro pharmacokinetics

**Figure 3:**
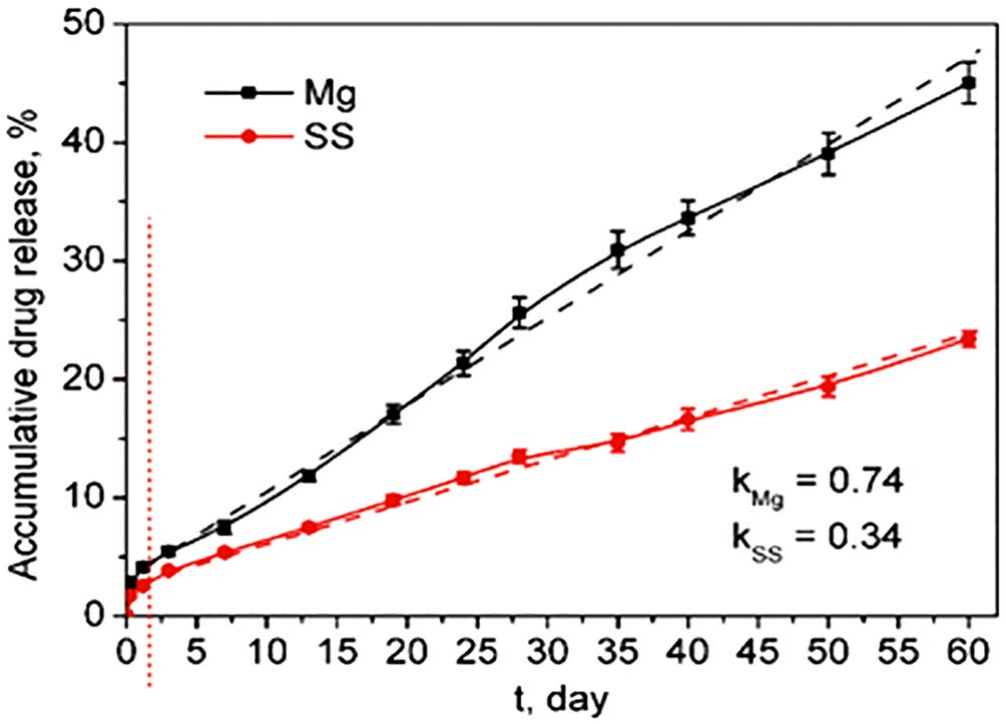
Drug release kinetic profiles of JDBM BRS and SS BRS.

The in vitro pharmacokinetics curves of JDBM BRS and SS BRS are shown in Fig. 3. The drug burst release ratios of the two groups were both lower than 5% within 1 day (marked by a dashed line in the figure). After the drug burst, the drug release displayed a linear relationship with time. The drug release rate constant calculated through y = kt was 0.74%/day for JDBM BRS and 0.34%/day for SS BRS. The drug release rate of JDBM BRS was much higher than SS BRS.

### Quantitative coronary angiographic results

As shown in Table 1, the reference vessel diameters were similar between the groups. Minimal luminal diameter behaved similarly pre-procedure and immediately after the procedure in both groups. Angiographic follow-up data were available in all experimental animals at 30, 90, and 180 days follow-up. The results showed that there was no significant difference in minimal luminal diameter between groups.

**Table 1:** Minimal lumen diameter of stented coronary artery segments measured by quantitative coronary angiographic (QCA).

	FIREHAWK	JDBM BRS	*P* value
Pre-procedure, n	4	4	
Reference vessel diameter, mm	2.63 ± 0.04	2.61 ± 0.05	0.6523
Minimal luminal diameter, mm	2.60 ± 0.08	2.56 ± 0.07	0.6443
30 day follow-up, n	4	4	
Minimal lumen diameter, mm	2.49 ± 0.13	2.40 ± 0.15	0.4577
90 day follow-up, n	4	4	
Minimal lumen diameter, mm	2.33 ± 0.15	2.18 ± 0.23	0.3455
180 day follow-up, n	4	4	
Minimal lumen diameter, mm	2.13 ± 0.11	2.01 ± 0.25	0.2165

Values are expressed as mean ± SD. n: number of stented arterial segments.

### OCT findings

**Figure 4:**
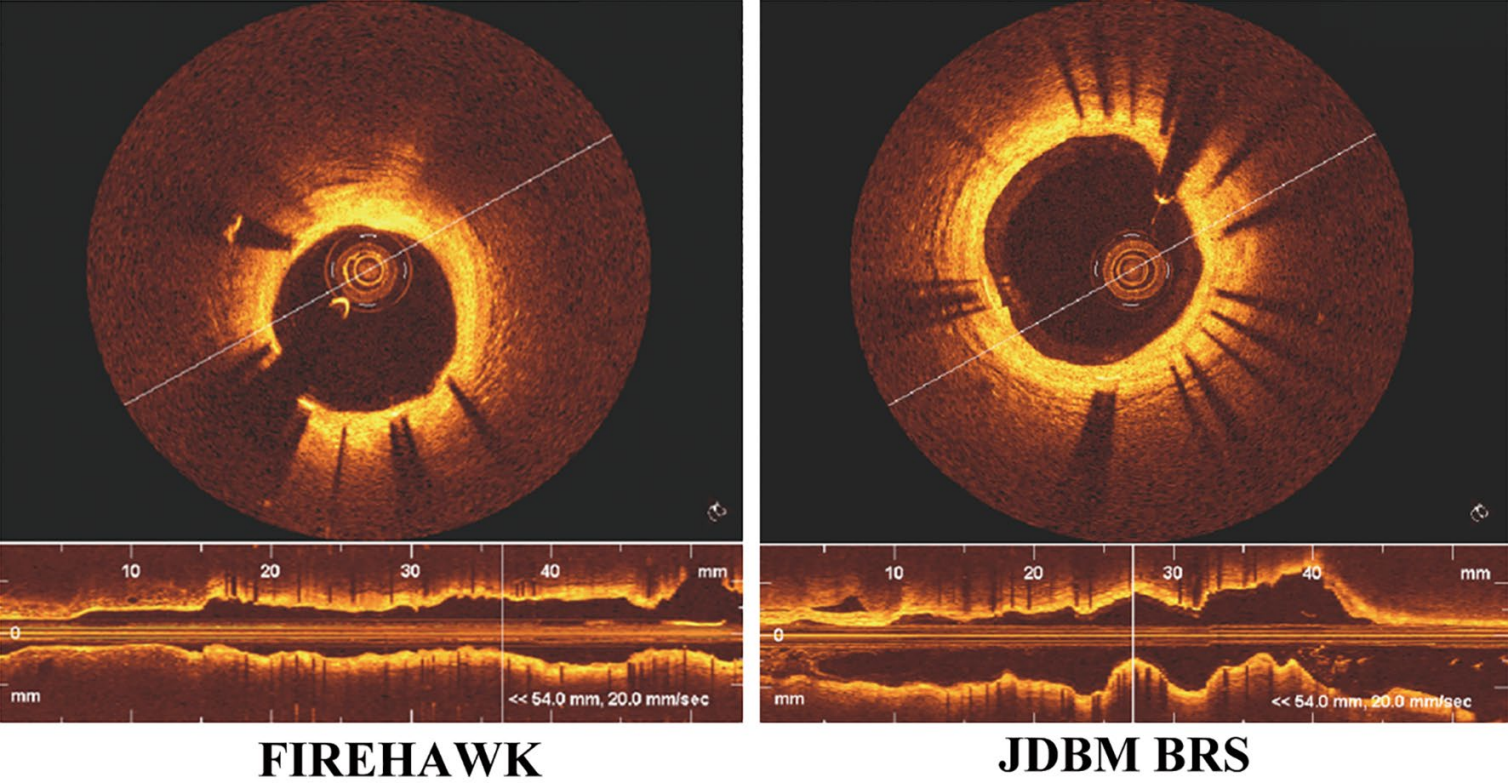
Representative OCT images of JDBM BRS and FIREHAWK stent in the porcine coronary artery at day 180 after stent implantation.

Sample OCT images in porcine coronary arteries implanted with the FIREHAWK and JDBM BRS stents for 180 days are shown in Fig. 4. At the cross-section level, there were no significant differences in mean lumen area (MLA), mean stent area (MSA), and mean neointimal area (NIA) between the FIREHAWK and JDBM BRS groups. No significant differences of uncovered struts, malapposed struts, and neovascularization (NV) were found between the two groups. No indices of thrombus were found in either FIREHAWK or JDBM BRS groups. These findings indicate the biosafety and efficacy of JDBM BRS in the present study (Table 2).

**Table 2:** OCT findings by cross section level.

	FIREHAWK (n = 4)	JDBM BRS (n = 4)	*P* Value
MLA (mm^2^)	6.5 ± 0.5	6.2 ± 0.2	0.470
MSA (mm^2^)	7.7 ± 0.5	7.4 ± 0.3	0.499
NIA (mm^2^)	1.2 ± 0.1	1.2 ± 0.2	0.809
Uncovered CS (%)	3.7 ± 1.3	4.4 ± 1.4	0.603
Malapposed CS (%)	0.8 ± 0.8	1.5 ± 1.0	0.436
Thrombus, n (%)	0 (0%)	0 (0%)	–
NV (%)	7.5	10.0	0.750

Values are expressed as mean ± SD. CS, cross section; MLA, mean lumen area; MSA, mean stent area; NIA, neointimal area; NV, neovascularization.

### Micro-CT study

**Figure 5:**
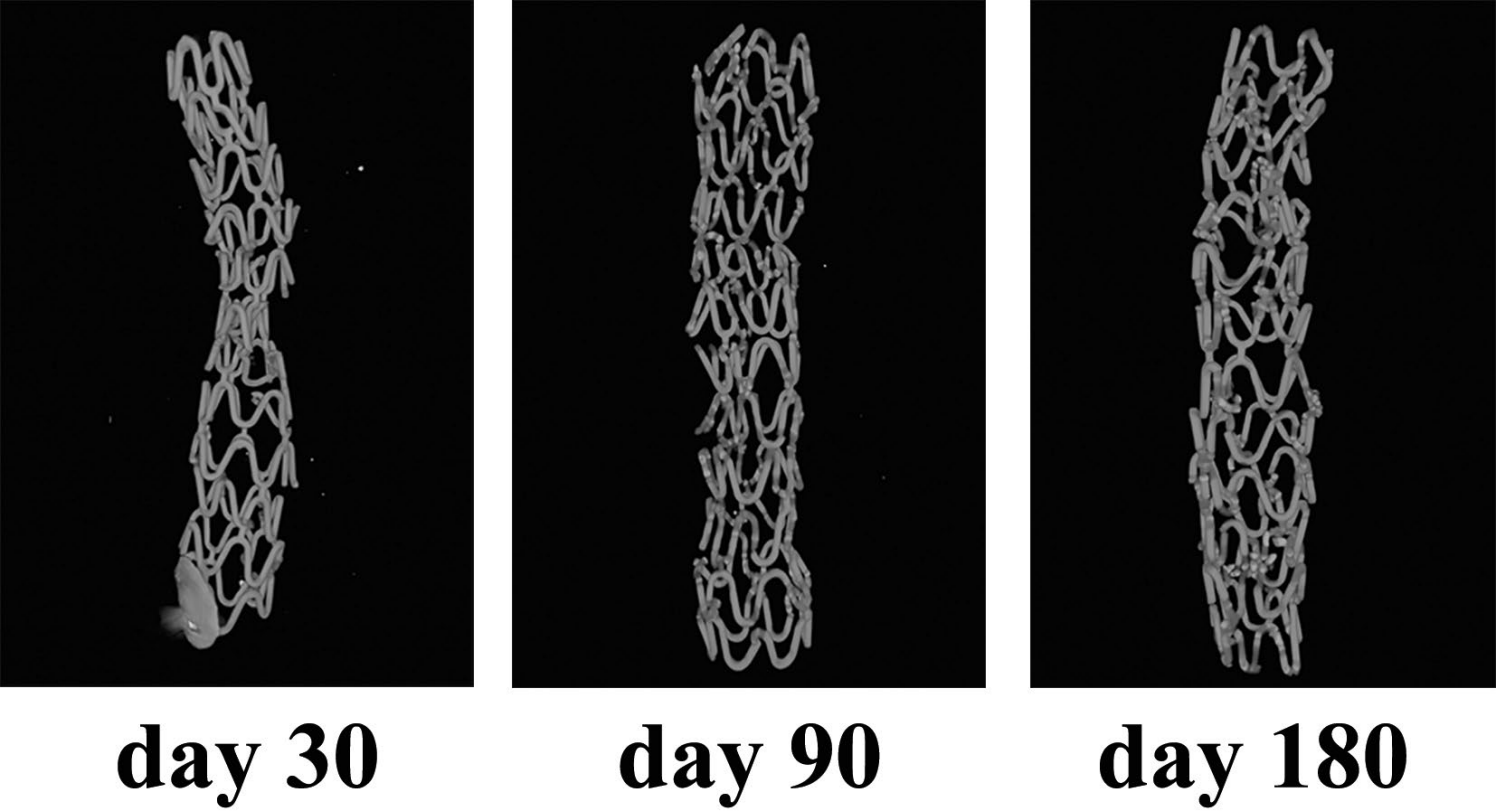
Micro-CT results of residual JDBM BRS at 30, 90, and 180 days post implantation. The stent still maintained good integrity at 30 days. Though some struts had transferred from the metal to degradation products, the main structure of the JDBM BRS stent remained intact at 90 and 180 days. The raw image data were reconstructed using the NRecon software V1.4.4.

To obtain the detailed morphology of JDBM BRS stents under biodegradation, JDBM BRS was harvested at 30, 90, and 180 days implantation and examined using micro-CTto evaluate the stent degradation through time, as shown in Fig. 5. At 30 days, the stent still maintained good integrity, indicating that the stent could still provide radial support to the vascular wall. At 90 days, the main structure of the JDBM BRS stent remained intact. Some struts had transferred from the metal to degradation products, which display as light gray in the image. Finally, at 180 days, JDBM BRS degraded more intensely but still maintained mechanical integrity. These outcomes revealed that the JDBM BRS stent supports vascular vessels effectively during the period prior to degradation post-implantation, and as anticipated began to lose its radial strength gradually with vascular physiologic reconstruction around 6 months.

### SEM analysis

**Figure 6:**
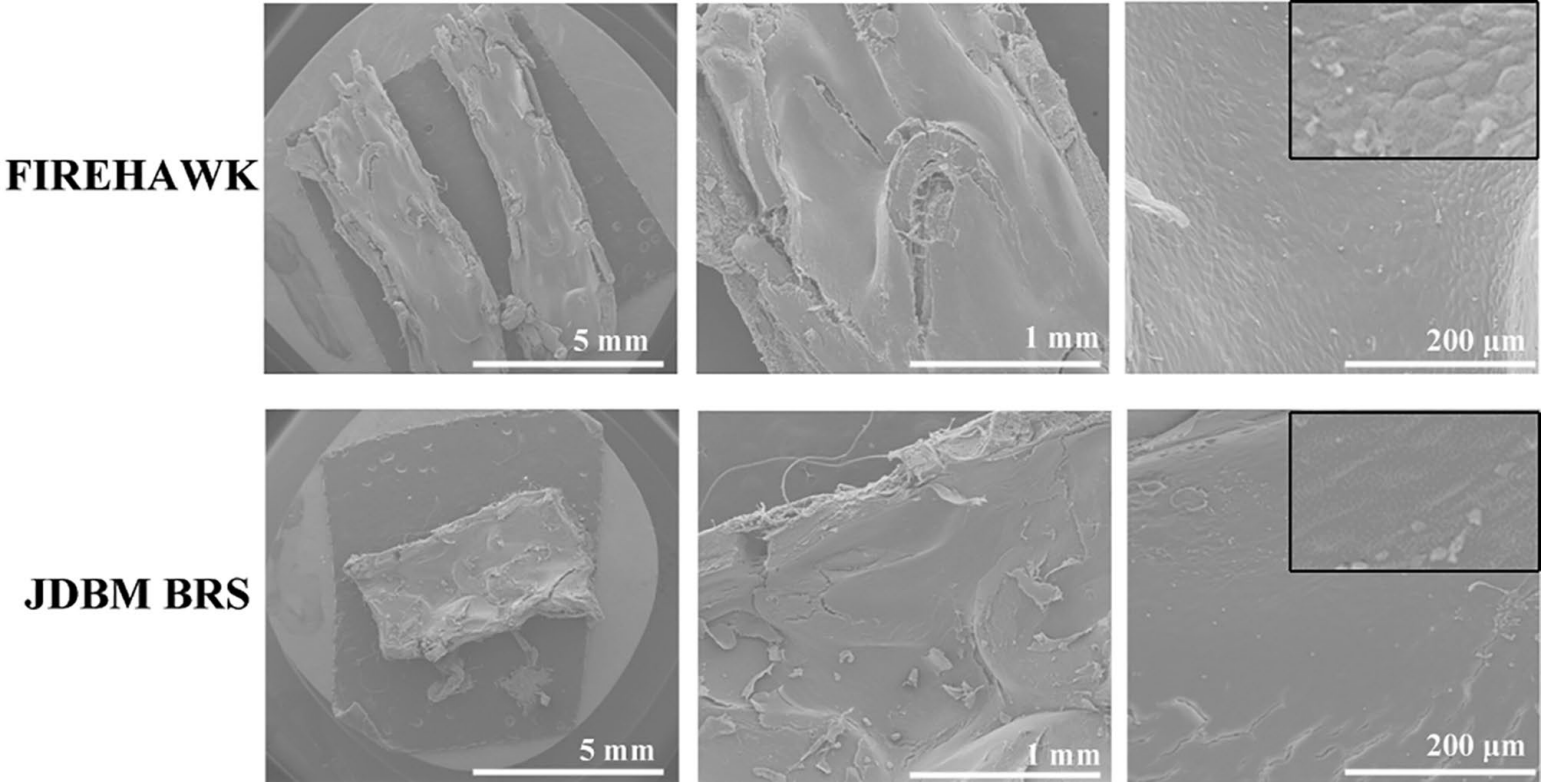
SEM images of JDBM BRS vs. FIREHAWK in the porcine coronary artery at 30 days post implantation.

At 30 days post stent implantation, the majority of stent surfaces showed nearly complete coverage in both groups. None of the groups showed obstructive luminal thrombi, which indicated that this novel JDBM BRS induced less inflammation within the vessel wall. Representative images are shown in Fig. 6.

### Histomorphometry and histology

**Figure 7:**
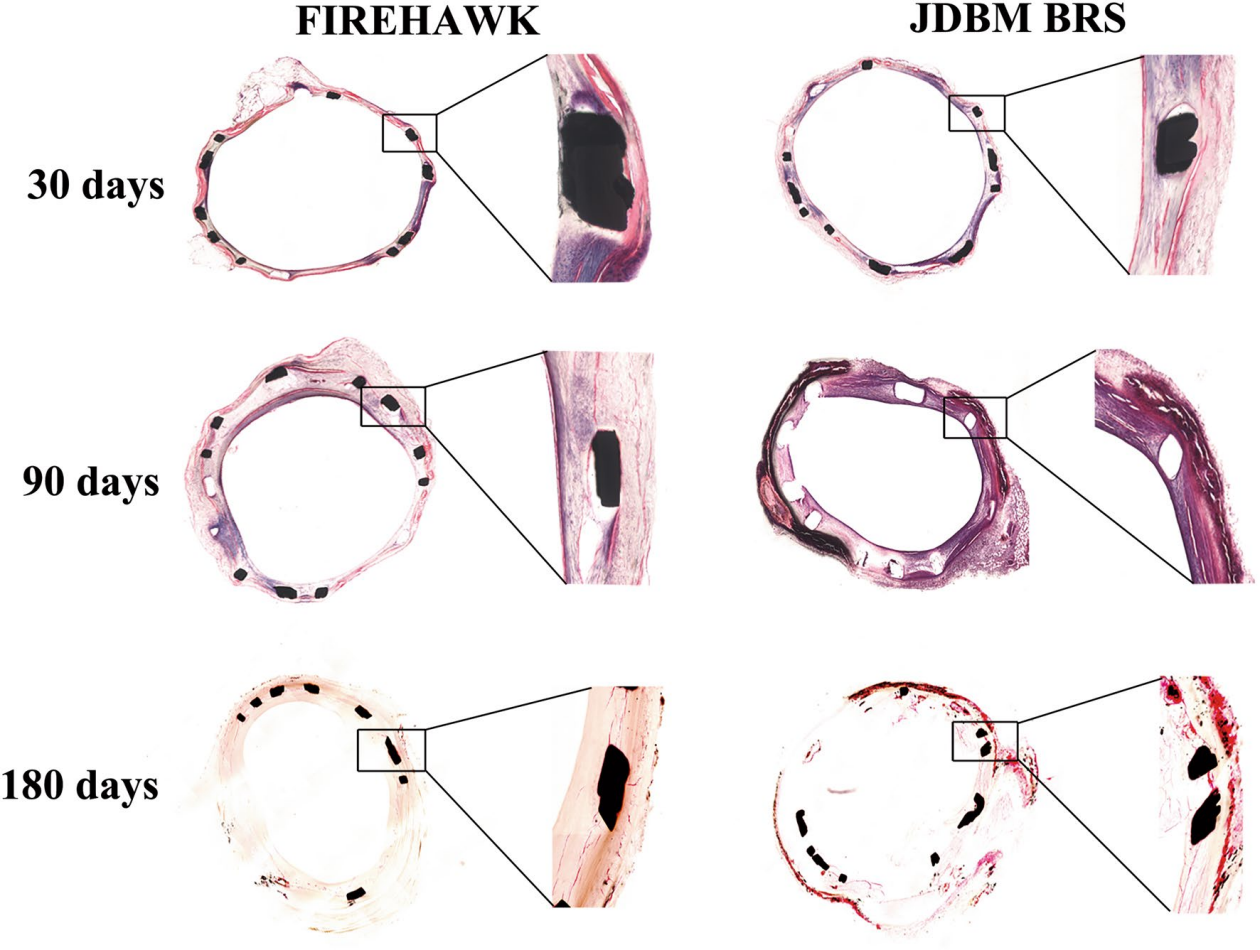
Representative histological images of JDBM BRS and FIREHAWK groups after implanted into porcine arteries for 30, 90, and 180 days. Sections are stained with Masson.

The in vivo safety and efficacy of JDBM BRS was evaluated by histological analysis of stented arteries in porcine. All animals underwent successful implantation of JDBM BRS and FIREHAWK rapamycin-eluting stent. There was no significant difference in the internal elastic membrane area, luminal area, neointimal thickness, neointimal area, and percent stenotic lumen among JDBMBRS and control groups, and injury and inflammation scores were similar in both groups, as shown in Table 3 and Fig. 7.

**Table 3:** Morphometry comparisons.

	FIREHAWK	JDBM BRS	*P* value
30 day follow-up, n	4	4	
Injury score	1.53 ± 0.11	1.57 ± 0.14	0.6776
Mean LA, mm^2^	4.56 ± 0.45	4.46 ± 0.63	0.6567
Mean IEM, mm^2^	5.67 ± 0.57	5.74 ± 0.77	0.5456
Mean NA, mm^2^	1.13 ± 0.44	1.35 ± 0.76	0.4477
Mean NT, mm	0.31 ± 0.09	0.45 ± 0.11	0.4876
Percent area stenosis, %	0.09 ± 0.02	0.13 ± 0.07	0.3676
Inflammation score	1.02 ± 0.14	1.15 ± 0.28	0.3577
90 day follow-up, n	4	4	
Injury score	2.04 ± 0.32	2.37 ± 0.44	0.6356
Mean LA, mm^2^	3.96 ± 0.45	3.75 ± 0.73	0.4567
Mean IEM, mm^2^	5.99 ± 0.88	5.84 ± 0.77	0.6657
Mean NA, mm^2^	1.96 ± 0.95	2.08 ± 1.11	0.3234
Mean NT, mm	0.51 ± 0.15	0.70 ± 0.23	0.3823
Percent area stenosis, %	0.15 ± 0.05	0.20 ± 0.08	0.3467
Inflammation score	0.89 ± 0.15	1.07 ± 0.28	0.2122
180 day follow-up, n	4	4	
Injury score	2.13 ± 0.31	2.39 ± 0.54	0.6745
Mean LA, mm^2^	3.66 ± 0.77	3.36 ± 0.73	0.3562
Mean IEM, mm^2^	6.38 ± 1.14	6.26 ± 1.57	0.3245
Mean NA, mm^2^	2.66 ± 0.83	2.86 ± 1.16	0.3649
Mean NT, mm	0.73 ± 0.21	0.92 ± 0.35	0.2556
Percent area stenosis, %	0.24 ± 0.05	0.29 ± 0.12	0.2345
Inflammation score	0.67 ± 0.23	0.87 ± 0.38	0.2187

Values are expressed as mean ± SD.

LA: average lumen area; IEM: average internal elastic membrane area; NA: average neointimal area; NT: average neointimal thickness; n: number of sections.

## Discussion

Drug-eluting BRS was designed to overcome the limitations associated with DES, such as a chronic local inflammatory reaction and late stent thrombosis in the treatment of CAD. Theoretically, ideal BRS should have adequate radial support for a period of about 6 months to limit recoil and constrictive remodeling. They should have as low of a crossing profile as possible and be flexible enough to allow delivery in more challenging and complex vascular lesions together with thin struts to limit the healing response. Finally, ideal BRS should be completely absorbed as soon as possible after the therapeutic period is over. Based on this theory, a first generation drug-eluting BRS was designed, with an efficacy profile similar to DES in de novo native coronary artery lesions^32–37^, indicating its long term advantages for the treatment of CAD.

BRS has thus undergone rapid development in recent years, and various types of polymers have been used. The most common polymer used is PLLA, which provides sufficient radial strength to the vessel. The radial strength is approximately 1200 mmHg directly after the implantation of the scaffold and it is as much as 800 mmHg after 1 year^9^. The degradation of PLLA occurs through hydrolysis of the ester bonds into small particles that are then phagocytosed by macrophages, thereby generating lactate. Lactate is subsequently converted into pyruvate and enters the Kreb’s cycle, where it is metabolized to CO_2_ and H_2_O^38^. The ABSORB bioresorbable vascular stent, the first PLLA-based drug-eluting BRS, demonstrated no difference in MACE when compared with traditional DES after 1-year clinical follow-up^33^. However, further investigations demonstrated the poor safety of these devices, which led Abbott Vascular to halt production of ABSORB^39^. Several limitations of PLLA should be addressed. First, overexpansion of PLLA may result in strut fractures because of the limitation in expansion and optimal scaffold apposition. Plus, reasonable device deployment is different for PLLA scaffolds because of the lack of radiopacity. Moreover, the behavior of PLLA scaffolds is limited in complex lesions such as bifurcations, calcification, and the presence of long or diffusely diseased lesions^40^.

Another polymer used in BRS technology is tyrosine polycarbonate, with its resulting copolymer a biodegradable polyester carbonate called poly(lactic acid-co-l-tyrosine). Following absorption, water, carbon dioxide, ethanol and iodinated tyrosine are the end products and excreted from the body^41^. Recently, REVA Medical developed a proprietary, inherently radiopaque polymer composed of tyrosine analogs and other natural metabolites that allow visualization using conventional angiography. The clinical evidence of the tyrosine polycarbonate polymer is promising but needs more research^42^.

Mg is an essential element required for several enzymatic reactions in the human body, and Mg-based scaffolds have been widely investigated due to their high mechanical strength. It is possible for Mg to form a scaffold with thinner struts, with degradation products of inorganic salts^16,43^. In comparison to PLLA, Mg shows better radial force, pushability and trackability^44^. Mg has been reported to have potential antithrombotic properties due to its electronegative charge during degradation^16,43,45,46^. Conversely, the reduction in ischemia–reperfusion injury using Mg has also been documented^47^, and Mg-mediated inhibition of endothelin-1 production is also known, which prevents endothelin-induced vasoconstriction^48,49^. In vitro tests of Mg-based BRS showed decreased smooth muscle cell proliferation and increased endothelial cell proliferation^50^. However, most Mg alloys exhibit excessive corrosion rates with complete biodegradation within 3 months, which leads to early vessel recoil and restenosis. This limits its clinical application, and the only reliable study available for Mg validation to date is BIOSOLVE II, where only 123 patients were treated, and any kind of complex lesion was excluded.

JDBM is a new type of patented Mg–Nd–Zn-Zr based alloy designed to circumnavigate the disadvantages of Mg; it has already exhibited good mechanical properties and a much slower degradation rate^17,18^. In the present study, we first immersed JDBM in HF to form MgF_2_ film for lower degradation of JDBM, and a PDLLA/RAPA coating was prepared for these stents to further reduce the degradation rate and prevent restenosis of the blood vessels by controlled and efficient rapamycin release to the coronary artery.

To explore the biocompatibility and specific biological functions of this novel JDBM BRS, in vitro and in vivo characterizations were carried out. In vitro biological functions, including effects on smooth muscle cells and drug release, were studied. Proliferation of smooth muscle cells in different groups was similar after 1 day. However, after 3 days, in contrast to the significant increase in controls, the cell density of the HF-JDBM group showed no obvious change, indicating cell proliferation was inhibited. When the PDLLA coating was added on the fluorinated JDBM, the cells in the PDLLA group grew as fast as the control group. As such, we assumed that the degradation rate of HF-JDBM was reduced by adding the PDLLA coating, and the high concentration of Mg ion release was avoided, which benefited cell proliferation^51^. When rapamycin was also added to the PDLLA coating, the cell density decreased in the PDLLA/RAPA group. This response is likely due to the inhibition of smooth muscle cell proliferation caused by rapamycin release. Rapamycin release plays an essential role in intimal hyperplasia inhibition and anti-thrombosis in drug-eluting stents, so JDBM BRS and SS DES with the same structure and drug coating were used to evaluate the drug release. The burst drug release rate was low for both stents. After 1 day, the drug release rate of JDBM BRS was gradually higher than SS DES, showing a linear relationship with time. This is because the degradation of Mg alloys can accelerate the rapamycin release, which is beneficial for lower restenosis risks. After 60 days, about 50% of the rapamycin had been released. The present in vitro studies reveal the great potential of JDBM BRS.

For in vivo evaluation in the present study, QCA, OCT, Micro-CT, SEM, and histomorphometry were performed. The vessels implanted with JDBM BRS and FIREHAWK were both able to receive complete endothelialization after 30 days. Further, there was no distinct thrombosis, intimal hyperplasia, restenosis, or inflammation during the longer implanted time to 180 days for both kinds of stents. Moreover, via micro-CT analysis, JDBM BRS showed sustained radial support after 180 days of implantation. These findings suggest that these stents can provide adequate support for 6 months or more. Compared with previous Mg alloy-based stent implantation results, most corners and connecting rods are broken only after 1 month^52^; therefore, adding the PDLLA/RAPA coating can reduce the degradation of JDBM stents dramatically, resulting in lower abnormal vasoconstriction risks and better vascular remodeling. However, JDBM BRS stent degradation characteristics over 180 days of implantation were not studied in the present investigation, leading to an incomplete understanding of the degradation characteristics of JDBM BRS. This is the main limitation of the present investigation, and delayed time analysis should be performed in future reports.

From the in vitro and in vivo results mentioned above, we conclude that after being loaded with a PDLLA/RAPA coating, JDBM BRS can inhibit smooth muscle cell proliferation and prevent intimal hyperplasia restenosis by controlling rapamycin release. Simultaneously, the degradation rates of JDBM stents are significantly reduced by the PDLLA coating and can thus provide radial support to the vascular wall over a period of 6 months. Therefore, JDBM BRS is a promising candidate for the treatment of coronary artery disease. Further investigations should be performed to reveal the exact degradation process of JDBM BRS.

## Conclusions

JDBM alloy-based rapamycin-eluting BRS could inhibit the proliferation of smooth muscle cells and prevent restenosis of the blood vessels by controllable and efficient rapamycin release while also exhibiting a moderate degradation rate. These results demonstrate that JDBM-based rapamycin-eluting BRS shave excellent potential as alternatives to the present DES for the treatment of coronary artery disease.
